# Effect of Size and Concentration of Copper Nanoparticles on the Antimicrobial Activity in *Escherichia coli* through Multiple Mechanisms

**DOI:** 10.3390/nano12213715

**Published:** 2022-10-22

**Authors:** Meng-Jiun Lai, Yue-Wern Huang, Hsuan-Chun Chen, Li-I Tsao, Chih-Fang Chang Chien, Bhaskar Singh, Betty Revon Liu

**Affiliations:** 1 Department of Laboratory Medicine and Biotechnology, College of Medicine, Tzu Chi University, Hualien 970374, Taiwan; 2 Department of Biological Sciences, Missouri University of Science and Technology, Rolla, MO 65409, USA; 3 Department of Clinical Laboratory Sciences and Medical Biotechnology, College of Medicine, National Taiwan University, Taipei 100229, Taiwan

**Keywords:** copper nanoparticles (CuNPs), bactericide, membrane leakage, reactive oxygen species (ROS), apoptosis, programmed cell death

## Abstract

Metal and metal oxide nanoparticles, including copper nanoparticles (CuNPs), display antimicrobial activities and are regarded as promising microorganism inhibitors. Here, we explored the antimicrobial activity of CuNPs in *Escherichia coli* (*E. coli*) using two particle sizes (20 and 60 nm) and five concentrations (1, 5, 10, 50 and 100 μg/mL). The result showed a concentration-dependent trend of bactericidal activities for both size groups, with 20 nm particles more effective than 60 nm particles at low concentrations. The membrane disruption caused by CuNPs was confirmed by electron microscopy, PI staining and protein leaking analysis. However, the results of reactive oxygen species generation and genomic DNA damage revealed that the size and concentration of CuNPs were factors affecting the induction of multiple bactericidal mechanisms simultaneously on different scales. Further results of annexin V-PI staining supported this hypothesis by showing the shifting composition of the early-, late- and non-apoptotic dead cells across the CuNP groups. Many CuNP treatment groups were rescued when four mammalian modulators—wortmannin, necrosulfonamide, Z-VAD-FMK, and SBI-0206965—were applied separately. The results suggest the possible existence of bacterial programmed cell death pathways in *E. coli* which could be triggered by CuNP treatments.

## 1. Introduction

The development of nanotechnology and nanoscience has flourished in the past two decades. Nanomaterials and nanoscale-sized related products have been applied in various fields including the semiconductor industry, optoelectronic engineering, biomedical technology, and agriculture, as well as the food sciences [1,2,3,4]. Based upon chemical composition, nanomaterials can be grouped into organics, inorganics, and hybrids [5]. Metal-based and metal oxide-based nanoparticles such as silver, gold, iron, zinc, zinc oxide, titanium oxide, and copper oxide are inorganic nanoproducts, and have given rise to concerns of potential hazards when employed in biomedical applications [6]. Numerous studies have suggested that metallic nanoparticles such as gold and copper nanoparticles (AuNPs; CuNPs) are able to serve as drug delivery carriers due to their easily functionalized surfaces, while the tunable optical property of metallic nanoparticles has advanced their applications to biosensing and therapeutic diagnosis [7,8,9]. Although some metallic nanoparticles are toxic, they can be included in wound dressing or instrument coatings as an antimicrobial agent [10,11,12].

Recently, the continuing emergence of antibiotic-resistant pathogenic organisms has been a growing problem for global health and food safety. The intrinsic antimicrobial properties of metallic nanoparticles show promise in the area and have been the subject of recent reviews [13,14]. Metallic nanoparticles have been shown not only to be effective against bacteria, without inducing bacterial resistance, but also against fungi and viruses [15,16,17]. Silver nanoparticles (AgNPs) and CuNPs have attracted attention, with their biomedical applications are either approved by the FDA or undergoing clinical trials [9,15,17]. AgNPs and CuNPs exhibit similar antibacterial activity towards *Staphylococcus aureus*, while AgNPs are slightly more effective against *Escherichia coli* (*E. coli*) [18]. However, this difference is offset by the lower cost of CuNPs [19].

The precise mechanisms involved in the antimicrobial activity of CuNPs are not clearly understood. Not only are the chemical properties and dosage important, but physical properties such as particle size (both primary and hydrodynamic), surface-area-to-volume ratio, and surface charge may also influence the antimicrobial activity [5,20]. The major focus of studies on the antimicrobial mechanisms of metallic nanoparticles has been in their propensity to generate reactive oxygen species (ROS) leading to oxidative stress in the biological milieu [13,14,21]. Oxidative stress leads to DNA damage, lipid oxidation and loss of membrane integrity in addition to oxidation of the functional proteins essential for cell growth [22]. Several nanoparticles have been demonstrated in the production of ROS, such as copper oxide, copper sulfate, Ag-Cu coated NPs, graphene oxide/copper oxide composites, and others [13,14]. There have been few studies on the generation of ROS by CuNPs [12], and in particular, there has been no study focusing on the integrative relationship between CuNP particle size, antimicrobial activity and molecular mechanism. Therefore, in this study, we aimed to establish the differential bactericidal effect on *E. coli* resulting from two sizes and five concentrations of CuNPs, and their mechanisms of action were also explored. Some possible programmed cell death pathways which had not been explored in prokaryotes were also revealed.

## 2. Materials and Methods

### 2.1. Preparation of Nanoparticles

Copper nanopowders of 25 nm and 60–80 nm were purchased from Sigma-Aldrich (Sigma-Aldrich, St. Louis, MO, USA). Stock solutions at 1 mg/mL of 25 nm (marked as 20 nm in article) and 60–80 nm (marked as 60 nm in article) CuNPs were capped with 1.0 mM of sodium dodecyl sulfate (SDS) (Sigma-Aldrich, St. Louis, MO, USA) as a dispersant, followed by an ultrasonic bath at 40 °C for 30 min to reduce aggregation of particles [23,24]. Working concentrations of CuNPs were used immediately. Silver nanoparticles (AgNPs; Sigma-Aldrich, St. Louis, MO, USA) containing sodium citrate as a stabilizer were prepared. Physiochemical properties of CuNPs were analyzed by a transmission electron microscope (TEM; H-7500; Hitachi, Tokyo, Japan), a liquid particle attractor (FlowVIEW, Hsinchu, Taiwan), a Flow AOI (FlowVIEW), and a Zetasizer Nano ZS (Malvern Instruments, Worcestershire, U.K.). CuNPs were prepared in double deionized water and then temperature-equilibrated at 25 °C for 120 s in a zeta cell. Sizes and zeta-potentials of CuNPs were measured using a Zetasizer Nano ZS and analyzed using Zetasizer software 6.30 (Malvern, Malvern, UK).

### 2.2. Cell Culture

*E. coli* (Migula) Castellani and Chalmers strain 25922 was purchased from the American Type Culture Collection (ATCC, Manassas, VA, USA) and cultured in Luria–Bertani (LB) broth (Becton, Dickinson and Company, Sparks, MD, USA) or LB agar (FocusBio, Miaoli, Taiwan). Bacteria were cultured at 37 °C in an aerobic condition. Human normal skin fibroblast WS1 (ATCC, Manassas, VA, USA; CRL-1502) was cultured in Minimum Essential Medium (MEM) (Gibco, Invitrogen, Carlsbad, CA, USA) supplemented with 10% (*v*/*v*) fetal bovine serum (Biological Industries; BI, Kibbutz Beit-Haemek, Israel) at 37 °C in a humidified incubator with 5% CO_2_.

### 2.3. Cell Viability Measurements in E. coli and Mammalian Cells

Unless stated otherwise, for each treatment group, an overnight culture of *E. coli* (about 10^9^ cells) was concentrated by centrifugation and resuspended in a working solution at a concentration of 10^9^ cells per mL.

To assess the impact on bacterial growth, for each treatment group, 10^8^ cells of *E. coli* were cultured in 1.3 mL of LB broth with various sizes and concentrations of CuNPs. The optical densities (O.D.) at an absorbance of 600 nm were measured hourly. In antimicrobial potential experiments, *E. coli* cells were treated with 20 or 60 nm of CuNPs at concentrations of 0 (mock), 1, 5, 10, 50, and 100 μg/mL at 37 °C for 24 h. The mock group contained the dispersant. Bacteria were treated with phosphate buffered saline (PBS) and 70% alcohol for 24 h as the negative and the positive controls, respectively. The colony numbers were determined by the standard colony counting method. For bacterial viability measurements, we employed the INT-dehydrogenase assay [25] and the PrestoBlue assay. The same treatments of CuNPs were applied as described above for *E. coli*. For the INT-dehydrogenase assay, 0.2 mg/mL of dehydrogenase activity indicator, iodonitrotetrazolium chloride (INT; Sigma-Aldrich, Saint Louis, MO, USA), was added to each treatment group. After incubating for 2 h at 37 °C and 200 rpm, dimethyl sulfoxide (DMSO; Sigma-Aldrich, Saint Louis, MO, USA) was added to each group for dissolution of violet formazan. Absorbance at 490 nm was measured by a Bio-Rad iMark microplate reader (Bio-Rad Laboratories, Hercules, CA, USA) while DMSO was served as a blank. The PrestoBlue assay was carried out according to the instruction manual of PrestoBlue^®^ Cell Viability Reagent (Invitrogen, Carlsbad, CA, USA), which was the same as previously described in the INT-dehydrogenase assay.

To evaluate the effects of modulators on bacterial death pathway, *E. coli* were pretreated with either 100 nM Z-VAD-FMK (marked as Z-VAD; Sigma-Aldrich, Saint Louis, MO, USA) for 30 min, 5 μM SBI-0206965 (marked as SBI; BioVision, Milpitas, CA, USA) for 2 h, 0.5 μM necrosulfonamide (NSA; Sigma-Aldrich, Saint Louis, MO, USA) for 1 h, or 100 nM wortmannin (marked as Wort; Abcam, MA, USA) for 30 min. The *E. coli* with and without the modulator pretreatment was then centrifuged, and resuspended in the CuNP suspensions, each at concentrations of 0, 1, 5, 10, 50, and 100 μg/mL, for 24 h. For a specific modulator pre-treated group, the CuNP working solution also contained the same modulator at the same concentration in the pre-treatment stage.

The fluorescent cell-viability reagent, PrestoBlue, was added and incubated for 2 h at 37 °C. Fluorescence at 590 nm measured by a Varioskan LUX multimode microplate reader (Thermo Fisher Scientific, Waltham, MA, USA) indicated the survival of cells [25].

To assess cytotoxicity of CuNPs in normal human cells, different amounts of 20 and 60 nm CuNPs were prepared in a serum-free medium with 30 min ultrasonic bath. Human normal skin fibroblast WS1 cells were treated with 20 or 60 nm CuNPs, each at concentrations of 0, 1, 5, 10, 50 and 100 μg/mL, at 37 °C for 24 h. Cells were treated with the serum-free medium and 100% DMSO for 24 h as the negative and the positive controls, respectively. The colorimetric 1-(4,5-dimethylthiazol-2-yl)-3,5-diphenylformazan (MTT; Sigma-Aldrich, Saint Louis, MO, USA) dye reduction assay was performed as previously described [26]. Cell viability was detected at the absorbance of 570 nm by a microplate reader.

### 2.4. Scanning Electron Microscopy

*E*. *coli* was grown to the log phase in LB broth, harvested by centrifugation, washed twice with PBS, and resuspended in PBS. Cells were divided into 12 groups and incubated with two sizes and a series of concentrations of CuNPs at 37 °C for 24 h. Negative control cells were treated with PBS while positive control cells were treated with 70% alcohol. All cells were fixed with 2.5% (*w*/*v*) glutaraldehyde in 0.1 M cacodylate buffer and 1% tannic acid (materials were kindly provided by Dr. S.-Y. Peng, Department of Biochemistry, Tzu Chi University, Hualien, Taiwan), thoroughly washed with 0.2 M cacodylate buffer, treated with 1% osmium tetroxide, and then dehydrated using a graded ethanol series (50%, 70%, 80%, 90%, 95%, and 100% ethanol). After critical-point drying and gold coating, the cells were examined under a scanning electron microscope (SEM; S-4700; Hitachi, Tokyo, Japan). The voltage of SEM was set at 15 kV and the magnification of images were 30,000×.

### 2.5. Transmission Electron Microscopy

*E. coli* were incubated with two sizes of various concentrations of CuNPs overnight, followed by centrifugation at 4000 × *g* for 10 min. Negative control cells were treated with PBS, while positive control cells were treated with 70% alcohol. Cell pellets were fixed with 2.5% (*w*/*v*) glutaraldehyde in 0.1 M cacodylate buffer and 1% tannic acid at 4 °C for 1 h, and then washed twice with 0.2 M cacodylate buffer. Osmium tetroxide at 1% was applied for secondary fixation, followed by washing in cacodylate buffer. A drop containing the bacteria was deposited onto a carbon-coated grid with 2% uranyl acetate, and the grids were examined using a TEM. The voltage of TEM was set at 80 kV and the magnification of images were 30,000×.

### 2.6. Membrane Leakage Assay

To assess the membrane leakage in bacteria, we used two different methods. One was by treating approximately 10^9^ of the cells with 1 mL 70% alcohol plus 0.1 % triton X-100. The other method was to suspend about the same amount of *E. coli* in 10 mL of LB broth and autoclave at 121 °C for 30 min. Cells treated with PBS were the negative control without membrane damage. Amounts of 20 nm and 60 nm CuNPs with a series of concentrations (0, 1, 5, 10, 50, 100 μg/mL) were added into cells followed by incubation at 37 °C for 24 h. Cells were twice washed with PBS and resuspended in 500 μL of FACS buffer, followed by flow cytometry analysis. *Propidium iodide* (PI) was added into each treated group for membrane leakage detection. The FL3 red fluorescence was measured by a Gallios Flow Cytometer (Beckman Coulter, Brea, CA, USA).

To understand the cytosolic protein leaking, *E. coli* cells were treated with two sizes of CuNPs in different concentrations for 24 h and spun down at 10,000 g for 10 min. An amount of 150 μL of supernatant in each group was pipetted with 150 µL of the Bradford protein assay reagent (Bio-Rad, Hercules, CA, USA) and mixed well with a shaker for 30 s, followed by 10 min incubation at room temperature. A series of bovine serum albumin solutions at 0, 200, 400, 600, 800, and 1000 μg/mL were used as the standard curve. The absorbance at 595 nm was measured by a microplate reader.

### 2.7. Reactive Oxygen Species Detection

The *E. coli* cells treated with different stress conditions such as 405 nm UV light for 3 h [27], 45 °C for 2 h [28], 4 °C for 2 h [28], and 3% H_2_O_2_ for 30 min [29], were the ROS-inducing positive controls. Cells suspended in LB medium at 37 °C was the negative control. Amounts of 20 nm and 60 nm CuNPs at concentrations of 0, 1, 5, 10, 50, 100 μg/mL were incubated with *E. coli* for 24 h. The treated cells were washed twice with PBS. The cell-permeant 2’,7’-dichlorodihydrofluorescein diacetate (H_2_DCFDA; Sigma-Aldrich, Saint Louis, MO, USA) was incubated with cells at a final concentration of 5 μM in PBS for 30 min at 37 °C [27,30]. The production of ROS in each group was analyzed in FL1 green fluorescence of a flow cytometer.

### 2.8. Chromosomal DNA Preparation

*E*. *coli* were divided into 12 groups and incubated with different sizes and concentrations of CuNPs at 37 °C for 24 h. Four other groups of cells were treated with 10 μg/mL AgNPs [31] at 37 °C for 3 h, 5 μg/mL ampicillin (Amp; Sigma-Aldrich, Saint Louis, MO, USA) [32] at 37 °C for 3 h, autoclaved at 121 °C for 30 min, and exposed to 70% alcohol for 3 h. Cells were then spun down and resuspended in the lysis solution containing 20 mg/mL proteinase K followed by the phenol/chloroform method for protein precipitation [33]. The aqueous solution was transferred to a new tube and the phenol/chloroform extraction was applied in an iterative fashion. Chromosomal DNA was precipitated with isopropanol followed by 70% alcohol. A NANO-100 micro spectrophotometer (HINOTEK Lab, Ningbo, China) was used to determine the concentration of chromosomal DNA in each group. DNA was analyzed by electrophoresis on a 1% agarose gel in 1 × TAE (40 mM of Tris-acetate and 1 mM of EDTA, pH8.0) buffer at 100 V for 25 min. Images were recorded by the Gel Doc EZ system with Image Lab^TM^ software (Bio-Rad, Hercules, CA, USA).

### 2.9. Identification of Apoptosis in E. coli

*E*. *coli* cells were cultured overnight and washed twice with PBS before being incubated with 10 μg/mL AgNPs or 5 μg/mL Amp for 3 h. Approximately 10^9^ bacterial cells in 10 mL LB broth were killed with an autoclave. In the apoptosis study, bacteria were incubated with two sizes of CuNPs at a series of concentrations for 24 h. Upon termination of experiments, cells were collected and washed once with 1 mL of cold, filtered 1 × PBS, followed by resuspension in 100 μL of annexin V-binding buffer (BioLegend, San Diego, CA, USA) and incubated with 5 μL of FITC-conjugated annexin V and 10 μL of PI solution for 15 min at room temperature [31]. Each sample was diluted by adding 400 μL annexin V-binding buffer and then placed on ice. The apoptosis results were analyzed by a Gallios Flow Cytometer. Autoclaving-treated cells were applied as the PI positive control, while 5 μg/mL Amp-treated cells were served as the Annexin V positive control.

### 2.10. Flow Cytometric Analysis

All staining assays were analyzed using a Gallios flow cytometer (Beckman Coulter, Brea, CA, USA) with a 525 BP filter (excitation at 488 nm and emission at 525 nm wavelength) on fluorescent channel 1 (FL1) for green fluorescent dye detections, and a 620 BP filter (excitation at 488 nm and emission at 617 nm wavelength) fluorescent channel 3 (FL3) for red fluorescent dye detections. The analyzed filter of the flow cytometer was set to the submicron scale for bacterial-sized measurements. Results are expressed as the percentage of the total cell population analyzed by the built-in Kaluza software.

### 2.11. Statistical Analysis

Data are expressed as mean ± standard deviation (SD). Unless specified, mean values and SDs were calculated from at least three independent experiments carried out in triplicate for each treatment group. Statistical comparisons were performed by one-way ANOVA, Kruskal–Wallis H test, and Student’s *t*-test, using statistical significance at *p* < 0.05.

## 3. Results

### 3.1. Bactericidal Activities of Two Sizes of Copper Nanoparticles

CuNP particles were characterized using TEM, a liquid particle attractor, and a Flow AOI (Figure S1a–c). The hydrodynamic sizes of 20 and 60 nm CuNPs were at 26.14 ± 8.37 and 66.95 ± 8.52 nm, respectively (Figure S1d,e).

**Figure 1:**
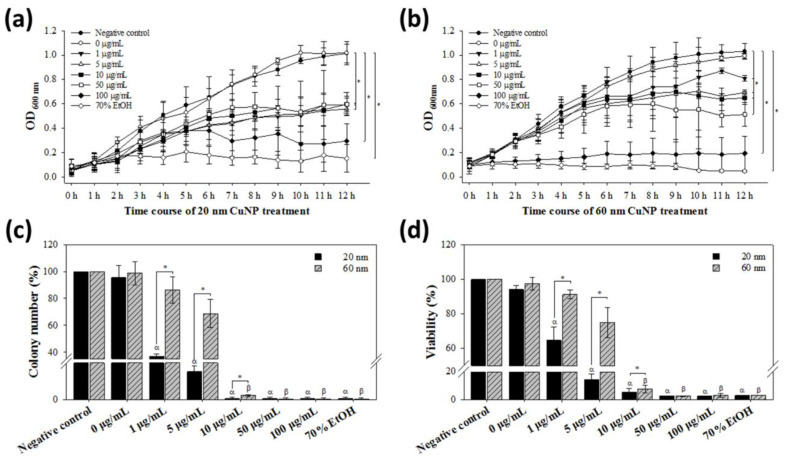
Bactericidal activities of CuNPs. *E. coli* were treated with different concentrations of CuNPs of 20 nm or 60 nm size. Bacteria treated with PBS or 70% alcohol were used as the negative and positive controls, respectively. (**a**,**b**) Determination of *E. coli* growth by optical density measurement. Cell growth curves from 0 to 12 h were analyzed with absorbance at 600 nm wavelength. Statistical calculation after treatment for 12 h was analyzed. (**c**) The percentage of colony formation of *E. coli* recovered after treatment with CuNPs for 24 h. (**d**) Bacterial viability analysis with PrestoBlue assay after treatment with CuNPs for 24 h. Bioactivities were determined at the emitting fluorescence of 590 nm. In both (**c**,**d**), one hundred percent value corresponds to the number in the negative control. Data are presented as mean ± SD from three independent experiments performed in triplicate for each treatment group. Statistical comparisons were performed by Student’s *t*-test; α and β indicate *p* < 0.05 for 20 and 60 nm CuNP treatments relative to the negative control, respectively, and * indicates *p* < 0.05 for comparisons between 20 and 60 nm of CuNPs in (**c**,**d**).

*E. coli* was examined for its susceptibility to CuNPs of both sizes at concentrations up to 100 μg/mL. In the growth curve experiments, the decline of bacterial growth treated by either size of CuNPs was both concentration- and time-dependent. At low concentrations (1, 5, and 10 μg/mL) of 20 nm CuNPs, cell growth started to be inhibited at 8 h, while high concentrations (50 and 100 μg/mL) started to inhibit growth at 7 h (Figure 1a). Significant inhibition of cell growth was observed at 12 h at all concentrations. In 60 nm CuNP treatment groups, the earliest growth suppression was observed at 100 μg/mL, but significant retardation of cell growth was detected in all concentrations at 12 h (Figure 1b).

We further assessed the bactericidal effect among the treated conditions by standard colony counting assay. Although the trend of concentration-dependent decrease in colony numbers was observed in both size groups, the bactericidal effect in the 20 nm treatment groups at low concentrations (1 and 5 μg/mL) of CuNPs was significantly higher than in the 60 nm groups (Figure 1c). The results of the bacterial viability were further confirmed using PrestoBlue assay (Figure 1d) and INT-dehydrogenase assay (result not shown); the outcome was similar to that from colony counting. On the other hand, no harm was detected in human primary skin cells WS1 after the treatment of 20 or 60 nm CuNPs across all concentrations (Figure S2). These results indicate that CuNPs possess concentration-dependent antibacterial activities, with 20 nm CuNPs more toxic than 60 nm CuNPs.

### 3.2. Effect of Nanoparticles on Bacterial Morphology and Membranes

**Figure 2:**
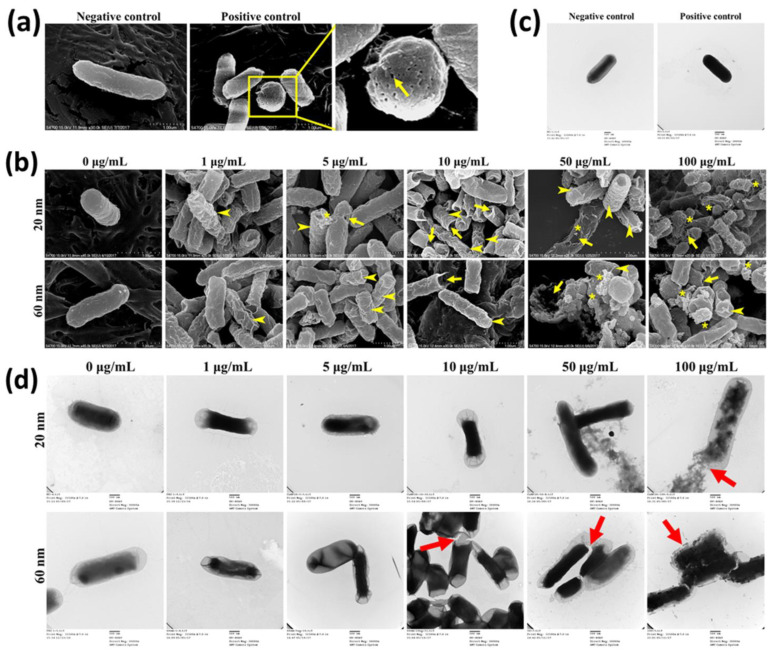
Electron microscopy images of *E. coli* treated with two sizes of CuNPs in different concentrations. Cells treated with PBS or 70% alcohol served as the negative control and positive control, respectively. (**a**,**b**) Live or dead cells observed via SEM. Images were obtained using a S-4700 SEM with a magnification of 30,000×. Arrow heads indicate the wrinkles of cell membranes; arrows mark the cracks on plasma membranes; stars illustrate the cytoplasmic components leaking from cells. Scale bar = 1 μm (**c**,**d**) Live or dead cells observed via TEM. Images were obtained using an H-7500 TEM with a magnification of 30,000×. Two percent of uranyl acetate was applied for the negative stain of cells followed by TEM imaging. Arrows indicate the membrane damage. Scale bar = 500 nm.

SEM and TEM analyses were carried out to understand concentration-dependent alteration of bacterial morphology induced by CuNPs (Figure 2). *E. coli* treated with PBS and 70% alcohol represented live cells (positive control) and dead cells (negative control), respectively. Several pinholes and fossettes on the surface of bacteria treated with alcohol were observed with SEM (Figure 2a). Dark morphologies with uranyl infiltration were recorded with TEM after alcohol treatment (Figure 2c). *E. coli* was treated with a series of concentrations of CuNPs. Shrunk and wrinkled cell surfaces were apparent characteristics of morphological damage (Figure 2b; arrowhead). Further, pinholes were seen in cells treated with low concentrations of 20 nm CuNPs (5 and 10 μg/mL) and 60 nm CuNPs (10 μg/mL) (Figure 2b; arrow). More severe damage was produced in higher concentrations of 20 and 60 nm CuNP treatments (50 and 100 μg/mL) and the leakage of intracellular components was revealed by spherical and filamentous deformation (Figure 2b; star). The TEM images suggest condensed intracellular matrixes induced by CuNP treatments (Figure 2d). The ruptured and fragmented cytoplasmic membrane may have led to leakage of cellular components (Figure 2d; arrow).

**Figure 3:**
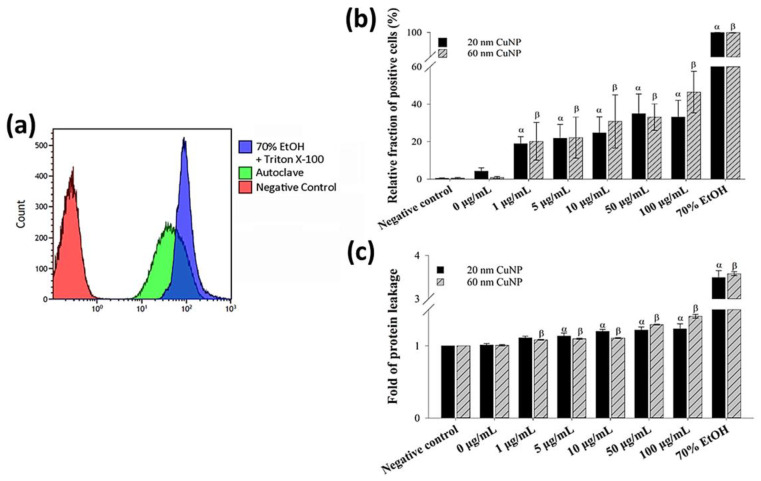
Membrane leakage detection in bacteria after being treated with two sizes of CuNPs in different concentrations for 24 h. (**a**) Flow cytometry profile of the bacterial population in control groups stained with PI dye. *E. coli* treated with PBS served as the negative control. *E. coli* treated with 70% alcohol plus triton X-100 or autoclaving served as positive controls. (**b**) Membrane permeability of *E. coli*. After CuNP treatment, PI staining was applied then analyzed using flow cytometry. (**c**) Cytoplasmic protein leakages in *E. coli* after CuNP treatment. Leaking proteins were collected from supernatants and detected by Bradford protein assay at an absorbance of 595 nm. Data are presented as mean ± SD from three independent experiments performed in triplicate for each treatment group. Statistical comparisons were performed by Student’s *t*-test; α and β indicate *p* < 0.05 for 20 and 60 nm CuNP treatments relative to negative control, respectively.

To further confirm the bacterial membrane leakage induced by CuNPs, a PI staining assay and a leaking protein detection were performed (Figure 3). PI is a cell membrane-impermeable fluorescent dye and only penetrates fissure membranes for DNA staining. Bacteria treated by either 70% alcohol plus triton X-100 or autoclaving serving as the positive control showed strong fluorescent intensities (Figure 3a). The degree of cytoplasmic protein leakage was further measured by the Bradford protein assay. The results of both analyses (Figure 3b,c) converged to show concentration-dependent membrane damage induced by CuNPs; however, no evident size effect was observed.

### 3.3. Influence of CuNPs upon Reactive Oxygen Species Production

**Figure 4:**
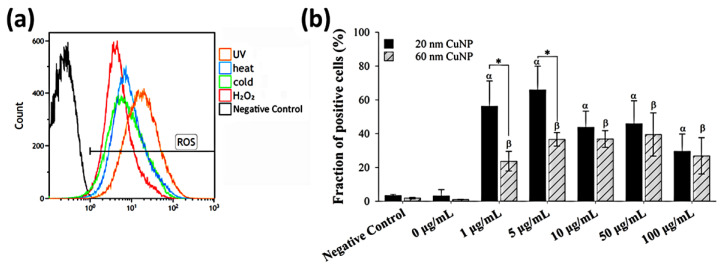
ROS generation in *E. coli* after CuNP treatment. (**a**) Flow cytometry analysis of ROS induced by various inducers in *E. coli*. Four groups of bacteria were subjected to the following conditions to induce ROS: 405 nm UV light for 3 h, 45 °C for 2 h, 4 °C for 2 h, and 3% H_2_O_2_ for 30 min. Bacteria cultured under normal conditions (37 °C, LB medium) and then washed with PBS served as the negative control. H_2_DCFDA was applied to bacteria to detect ROS. (**b**) Fractions of fluorescent positive cells in different CuNP treatments. Bacteria were treated with two sizes of CuNPs in different concentrations for 24 h and then stained with H_2_DCFDA. Fluorescence was detected by flow cytometry. These data are presented as mean ± SD from five independent experiments performed in triplicate for each treatment group. Statistical comparisons were performed by Student’s *t*-test; α and β indicate *p* < 0.05 for 20 and 60 nm CuNP treatments relative to the negative control, respectively, and * indicates *p* < 0.05 for the comparisons between 20 and 60 nm of CuNPs.

As many metallic nanoparticles and metal oxide nanoparticles or their released ions were known to be able to generate ROS leading to the antimicrobial processes, we used H_2_DCFDA combined with flow cytometry to assess ROS production induced by CuNPs in *E. coli* (Figure 4). Bacterial cells treated with either UV light, heat, cold, or 3% H_2_O_2_ were used as positive controls, in which strong fluorescence was detected indicating elevated ROS (Figure 4a). The results (Figure 4b) showed that compared with PBS (negative control), significant fractions of ROS-positive cells were observed in all groups treated with CuNPs of both sizes. However, in contrast with the bactericidal effect and membrane disruption, no concentration-dependent trend was shown for ROS production in *E. coli* after treatment by either size of CuNPs. Additionally, the fraction of positive cells in the 1 and 5 μg/mL groups was much higher for the particle size of 20 nm, relative to 60 nm.

### 3.4. DNA Damage and CuNP Treatment

**Figure 5:**
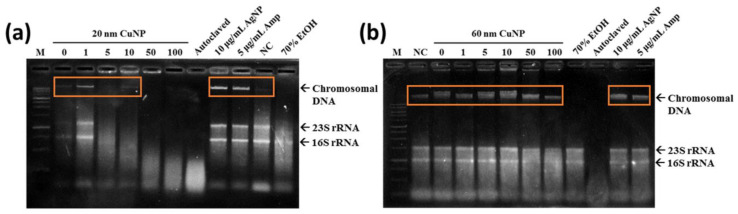
Total genomic DNA analysis of *E. coli* via electrophoresis. Chromosomal DNA damages were detected in bacteria treated with 20 nm CuNPs (**a**), or 60 nm CuNPs (**b**), in a series of concentrations (0, 1, 5, 10, 50, 100 μg/mL) for 24 h followed by genomic DNA extraction. Ten μg/mL AgNPs and 5 μg/mL Amp were incubated with bacteria for 3 h to induce condensation of DNA and early apoptosis in *E. coli*. Autoclaving and 70% alcohol treatments were applied to induce acute cell death. PBS treatment was used as a negative control (NC) in the study of cell survival and chromosomal DNA integrity. One kb DNA ladder was loaded as the marker (M). The arrows indicated the positions of chromosomal DNA, 23S rRNA, and 16S rRNA. Orange frames marked the condensed DNA. DNAs were analyzed by agarose-based gel electrophoresis, and a Gel Doc EZ imaging system was used to obtain images.

Oxidative stress has been associated with lipid, protein, and DNA damage. Therefore, we evaluated the genomic DNA damage in *E. coli* associated with CuNP treatment. Exposure of *E. coli* to 5, 10, 50 and 100 μg/mL of 20 nm CuNPs generated a concentration-dependent increase in DNA smearing and disappearance, indicating DNA damage and degradation (Figure 5a). However, for *E. coli* treated with 1 μg/mL of 20 nm CuNPs or any concentration of 60 nm CuNPs, instead of DNA smearing or disappearing, a condensed band was observed (Figure 5a,b; orange frames). This was similar to the electrophoresis pattern seen in the DNA of the cells treated with Amp or AgNPs, both of which were thought to be early apoptosis inducers in bacteria [31,32]. We speculated that the cell death induced in these groups was probably through similar mechanisms as Amp and AgNPs. Additionally, the patterns of ROS production in the groups treated with either size of CuNPs (Figure 4) were dissimilar to the patterns of DNA damage. The difference may imply mechanism(s) other than DNA damage being involved in the antibacterial activities of 20 and 60 nm CuNPs.

### 3.5. CuNP-Induced Apoptosis in E. coli

According to observations of the DNA damage patterns in gel electrophoresis (Section 3.4), we further evaluated the cell death induced by CuNPs using FITC-conjugated annexin V-PI double staining and flow cytometric analysis. Annexin V can bind to exposed phosphatidylserine on the surface of a dying cell as an apoptotic marker, while PI, a membrane-impermeant nucleic acid intercalator, is an indicator of dead cells. This double-staining approach is commonly used to detect apoptosis in eukaryotic cells, and has also been employed to study bacterial death induced by antibiotics [32] and AgNPs [31].

**Figure 6:**
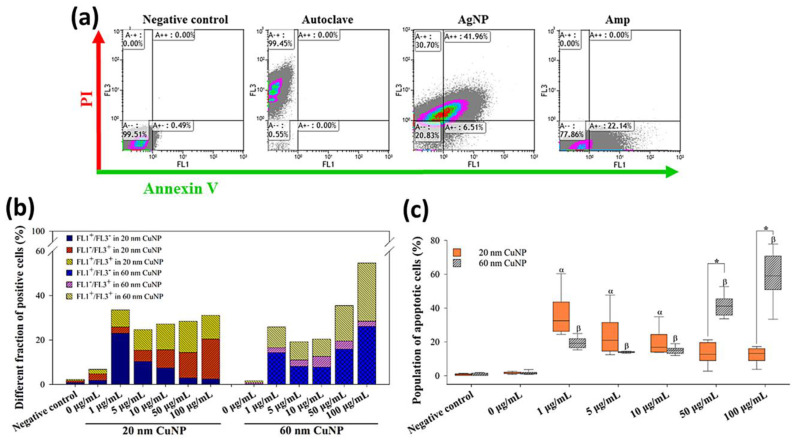
Flow cytometry analysis of annexin V-PI staining in *E. coli* after CuNP treatment. (**a**) *E. coli* was treated with PBS (negative control), autoclaving, 10 μg/mL AgNPs, and 5 μg/mL Amp, followed by annexin V-PI staining. FL1 and FL3 were regarded as annexin V and PI signals. (**b**) Different fractions of positive cells from quadrants. Bacteria were treated with two sizes of CuNPs in different concentrations for 24 h. Bacteria treated with PBS served as the negative control. Fluorescent intensity was acquired by flow cytometry, and single positive (FL1^+^/FL3^−^; FL1^−^/FL3^+^) as well as double positive (FL1^+^/FL3^+^) signals were calculated. (**c**) Statistical analysis of apoptosis bacterial cells which were represented by the sum of percentages from FL1^+^/FL3^−^ (early stage) and FL1^+^/FL3^+^ (late stage). Data were presented as mean ± SD from six independent experiments performed in triplicate for each treatment group. Data were analyzed by the Kruskal–Wallis H test followed by Dunn’s *post hoc* test; α and β indicate *p* < 0.05 for 20 and 60 nm CuNP treatments relative to the negative control, respectively. Statistical difference between 20 and 60 nm CuNP treatments was set at *p* < 0.05 (*) according to the paired *t*-test.

Adopting the patterns as described in [31], that annexin V-positive/PI-negative (FL1^+^/FL3^−^) and annexin V-positive/PI-positive (FL1^+^/FL3^+^) cells are apoptotic bacteria in early and late stages, respectively, then annexin V-negative/PI-positive (FL1^-^/FL3^+^) cells represent dead bacteria in the non-apoptotic phase. In the results shown in Figure 6a, about 99.45% of *E. coli* were dead cells with non-apoptotic features (FL1^−^/FL3^+^) in autoclaving treatments, while high fractions of cells were detected as apoptotic cells (FL1^+^) in AgNP or Amp treatments (48.47% and 22.14%, respectively) (Figure 6a). In the groups treated with 20 nm CuNPs, a high fraction of apoptosis (FL1^+^; Figure 6c), more specifically, early apoptosis (FL1^+^/FL3^−^), was seen at low concentrations (Figure 6b). The population of apoptotic cells in the 1 μg/mL-treated *E. coli* reached about 30% (Figure 6c), higher than the level seen in Amp-treated cells (22.14% in Figure 6a). As the concentration of 20 nm CuNPs increased, cell fractions shifted from the early apoptotic (FL1^+^/FL3^−^) to the non-apoptotic (FL1^−^/FL3^+^) phase (Figure 6b). In comparison, in the groups treated with 60 nm CuNPs, the fractions of apoptotic cells (FL1^+^; Figure 6c) at 1, 5 and 10 μg/mL were about 20%, and surged to high levels at 50 and 100 μg/mL treatments (41.3% and 59.1%, in 50 and 100 μg/mL, respectively; Figure 6b).

The collective outcome of the ROS generation, genomic DNA gel electrophoresis and annexin V-PI double staining suggest that multiple death mechanisms can be triggered within the context of concentration and size of CuNPs.

### 3.6. Multiple Bacterial Death Mechanisms of CuNPs Revealed by Four Modulators

Several bacterial programmed cell death systems have been proposed with features sharing a certain degree of similarity to eukaryotic systems [34]. In this study, we chose four mammalian cell-death modulators to further characterize the bactericidal death mechanisms of CuNPs.

Z-VAD is a compound that binds to the proteins of the caspase family and blocks the initiation of apoptosis [35]. NSA suppresses necroptosis, a programmed necrotic cell death, by inhibition of mixed lineage kinase domain-like pseudokinase (MLKL) [36]. The small-molecule ULK1 inhibitor, SBI, is highly selective to serine/threonine autophagy-initiating kinases ULK1 [37]. Wort blocks the formation of autophagosomes by disruption of the class III phosphatidylinositol 3-kinase (PI3K) [38].

**Figure 7:**
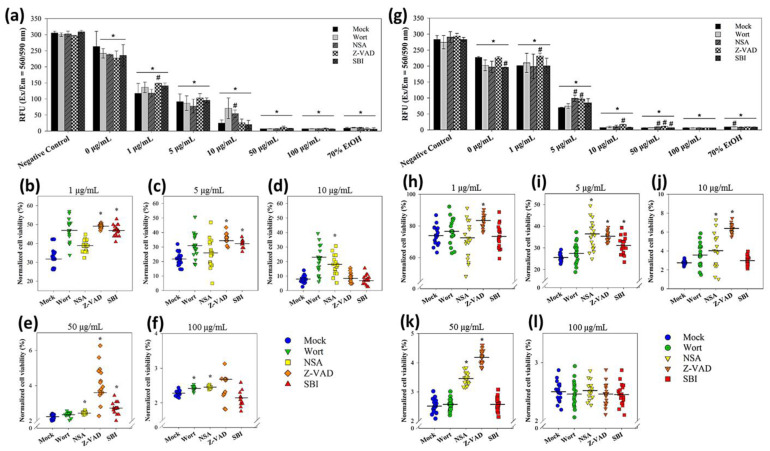
Mechanistic studies of bacterial death due to exposure to 20 or 60 nm CuNPs. (**a**) Viability analysis in the treatments of 20 nm CuNPs with various modulator interventions. Bacteria were treated with PBS (negative controls), 0, 1, 5, 10, 50, 100 μg/mL of 20 nm CuNPs in the absence (mock) or presence of wortmannin (Wort), necrosulfonamide (NSA), Z-VAD-FMK (Z-VAD), and SBI-0206965 (SBI). Cell viabilities were determined by PrestoBlue assay. Relative fluorescence units were acquired at the 590 nm emission from 560 nm excitation. Statistically significance was set at *p* < 0.05 (* vs. negative control and # vs. mock). (**b**–**f**) Normalized cell viabilities in the treatments of 20 nm CuNPs corrected for modulators in the negative control. Data are presented as mean ± SD from eighteen independent experiments performed in triplicate for each treatment. Differences from the mock were considered significant at *p* < 0.05 (*) using one-way ANOVA. (**g**–**l**) For comparison, the same viability analysis and normalized cell viability analysis were conducted in the treatments of 60 nm CuNPs with various modulator interventions. Data in (**h**–**l**) are presented as mean ± SD from nineteen independent experiments performed in triplicate for each treatment group.

The decreases in cell viability were concentration-dependent in both sizes of nanoparticles in the absence or presence of the modulators (Figure 7a,g). Statistics suggested that *E. coli* cells exposed to 1 and 5 μg/mL of 20 nm CuNPs were rescued using Z-VAD (Figure 7b,c). For the 60 nm CuNP treatment groups, Z-VAD was able to rescue *E. coli* at all concentrations except for 100 μg/mL (Figure 7h–l). Using NSA, increased cell viability was observed in the 10, 50, and 100 μg/mL 20 nm CuNP treatment groups (Figure 7d–f). Significant improvement of cell viability by NSA was detected in the cells treated in 5, 10, and 50 μg/mL of 60 nm CuNPs (Figure 7i–k). SBI rescued the cells treated with 1, 5, and 50 μg/mL of 20 nm CuNPs (Figure 7e,f). SBI-dependent cell rescue only existed in the 5 μg/mL 60 nm CuNP group (Figure 7i). Wort was only able to increase cell viability in the 100 μg/mL 20 nm CuNP group.

The data suggest that the death process which could be rescued by Z-VAD was a type of bacterial death under the treatment with low concentrations of 20 nm CuNPs and most tested concentrations of 60 nm CuNPs. The death pathway targeted by NSA became one of the dominant mechanisms of death induced at high concentrations of 20 nm CuNPs, and played a considerable role at 5, 10 and 50 μg/mL of 60 nm-CuNPs. The death pathways which could be rescued by SBI or wort were evidently triggered at certain conditions of CuNP treatment.

## 4. Discussion

In this study, both 20 nm and 60 nm CuNPs effectively exerted antimicrobial action, but exhibited negligible cytotoxicity in human normal skin cells (Figure 1 and Figure S2). We employed several approaches to examine and compare the bacterial death triggered by both sizes of CuNPs at varying concentrations. Metallic nanoparticles and their oxide composites are regarded as bactericides resulting from ROS attack [13,14,15,16]. Smaller nanoparticles have been suggested to produce a higher degree of ROS due to their larger surface area to volume ratio, leading to more severe cellular damage [13,20]. Our study showed that both 20 nm and 60 nm CuNPs elevated cellular ROS in *E. coli* (Figure 4b); however, it was only at low concentrations (1 and 5 μg/mL) that 20 nm nanoparticles showed statistically higher production of ROS relative to 60 nm groups. Additionally, no rising trend in either size group was seen when the concentration increased, as observed in the bactericidal analysis. Our subsequent analysis of DNA gel electrophoresis, annexin V-PI staining and modulator rescue experiments collectively suggested that bactericidal activities of CuNPs were influenced by both particle size and concentration through multiple mechanisms (Figure 6 and Figure 7).

Mammalian cells and prokaryotes share some proteins with similar functions [39,40]. Cytophysiology such as endocytosis, cytoskeleton movement, and programmed cell death were considered as unique traits in eukaryotes, but recently have been identified in prokaryotes [40,41,42,43]. For instance, the MazEF death pathway in *E. coli* induces membrane depolarization as well as DNA fragmentation, similar to caspase-activation, and was called apoptotic-like death (ALD) [41]. When we applied mammalian cell-death modulators to study the antimicrobial mechanisms induced by CuNPs, different degrees of enhanced survival were observed.

Z-VAD is a pan-caspase inhibitor and can block the initiation of apoptosis in eukaryotic cells [35]. Our results showed that it was able to rescue *E. coli* under most CuNP treated conditions, which were revealed by annexin V-PI staining to be composed of a high fraction of apoptotic cells. In the NCBI protein database, there are proteins (such as WP_000709408) from *E. coli* annotated as caspases and highly similar to the human mucosa-associated lymphoid tissue lymphoma translocation protein 1 isoform b: NP_776216 and AAG38589 (Figure S3a). These caspase-like proteins in *E. coli* are good candidates for the study of the roles of such proteins in bacterial death triggered by CuNPs.

SBI is a potent and selective inhibitor to human threonine-protein kinase ULK1 (NP_003556), an autophagy initiator [37]. Many proteins from *E. coli* were shown to share the similar catalytic domain of ULK1 via blast search (Figure S3b). Wort is also an autophagy inhibitor, and is able to covalently interact with the catalytic subunit of PI3K [38,44]. PI3K is categorized into classes I, II, and III based on their sequences and substrate specificity; they have generally been considered to exist in eukaryotes [45]. Individual catalytic domains of variants from different classes of PI3Ks were used as the query to blast (blastp and tblastn) searches against sequence databases. Though several bacterial proteins were suggested to contain PI3K-like catalytic domains by the blast result, none were from *E. coli* (data not shown). In bacteria, the toxin–antitoxin system was suggested to be functionally analogous to the autophagic cell death process [46]. Though the connection between the toxin-antitoxin system and the death pathways that SBI- or Wort can inhibit is not clear, our data suggest that that the death process targeted by SBI or Wort might be one of the cell death mechanisms in *E. coli*, and can be triggered by CuNPs in certain combinations of size and concentration.

The death pathway targeted by NSA played an important role when *E. coli* was treated by 20 nm CuNPs at high concentrations and 60 nm at low concentrations (Figure 7). In our annexin V-PI staining experiment, a good fraction of bacteria in these groups were in the non-apoptosis dead phase. In human cells, NSA can suppress necrotic programmed cell death by binding to the 4HB domain of MLKL (NP_689862.1) [47,48]. However, when we searched NP_689862.1 against *E. coli* protein sequences, no significant similar candidates were found. The only blast hit in bacteria was the hypothetical protein from *Legionellaceae bacterium* (MAZ77480) (data not shown). Although we could not identify the possible domains targeted by NSA in *E. coli* through our sequence-based search, we cannot rule out their existence. On the other hand, bacterial “autolysis” shares morphological features with programmed necrosis [46]. The connection between bacterial autolysis, non-apoptosis dead pathways, and the death processes targeted by NSA, is worthy of exploration.

## 5. Conclusions

CuNPs are capable of bacterial growth inhibition, membrane disruption, DNA damage, and oxidative stress induction in bacteria. We found particle size- and concentration-dependent triggering of multiple bacterial death mechanisms via exposure to CuNPs. Using eukaryotic modulators to explore the possible underlying death pathways in *E. coli* after CuNP treatment further suggests that a certain degree of similarity exists between cell death systems in prokaryotes and eukaryotes. Our study emphasizes the poor understanding of cell death in bacteria, and the need to conduct additional research on signal pathways and bactericidal targets. Collectively, our findings may be used to advance the development of antimicrobial strategies.

## Supplementary Materials

The following supporting information can be downloaded at https://www.mdpi.com/article/10.3390/nano12213715/s1: Figure S1: Size determination of CuNPs; Figure S2: Cytotoxicity of 20 and 60 nm CuNPs in mammalian cells; Figure S3: Sequence alignment of modulator active domains in *E. coli*.
