# Supplementary material for: Effect of Size and Concentration of Copper Nanoparticles on the Antimicrobial Activity in Escherichia coli through Multiple Mechanisms
Source: Nanomaterials (Basel). 2022 Oct 22;12(21):3715. doi: 10.3390/nano12213715 (PMC9656174; doi:10.3390/nano12213715)
Supplement: Supplementary file 1 [file nanomaterials-12-03715-s001.zip › nanomaterials-1917981-supplementary.pdf]

Supplementary Materials

Effect of Size and Concentration of Copper Nanoparticles on the Antimicrobial Activity in *Escherichia coli* through Multiple Mechanisms

Meng-Jiun Lai [1], Yue-Wern Huang [2], Hsuan-Chun Chen [1], Li-I Tsao [3], Chih-Fang Chang Chien [3], Bhaskar Singh [1] and Betty Revon Liu [1,*]

[1] Department of Laboratory Medicine and Biotechnology, College of Medicine, Tzu Chi University, Hualien 970374, Taiwan
[2] Department of Biological Sciences, Missouri University of Science and Technology, Rolla, MO 65409, USA
[3] Department of Clinical Laboratory Sciences and Medical Biotechnology, College of Medicine, National Taiwan University, Taipei 100229, Taiwan
* Correspondence: brliu7447@gms.tcu.edu.tw; Tel.: +886-3-856-5301 (ext. 2326); Fax: +886-3-857-1917

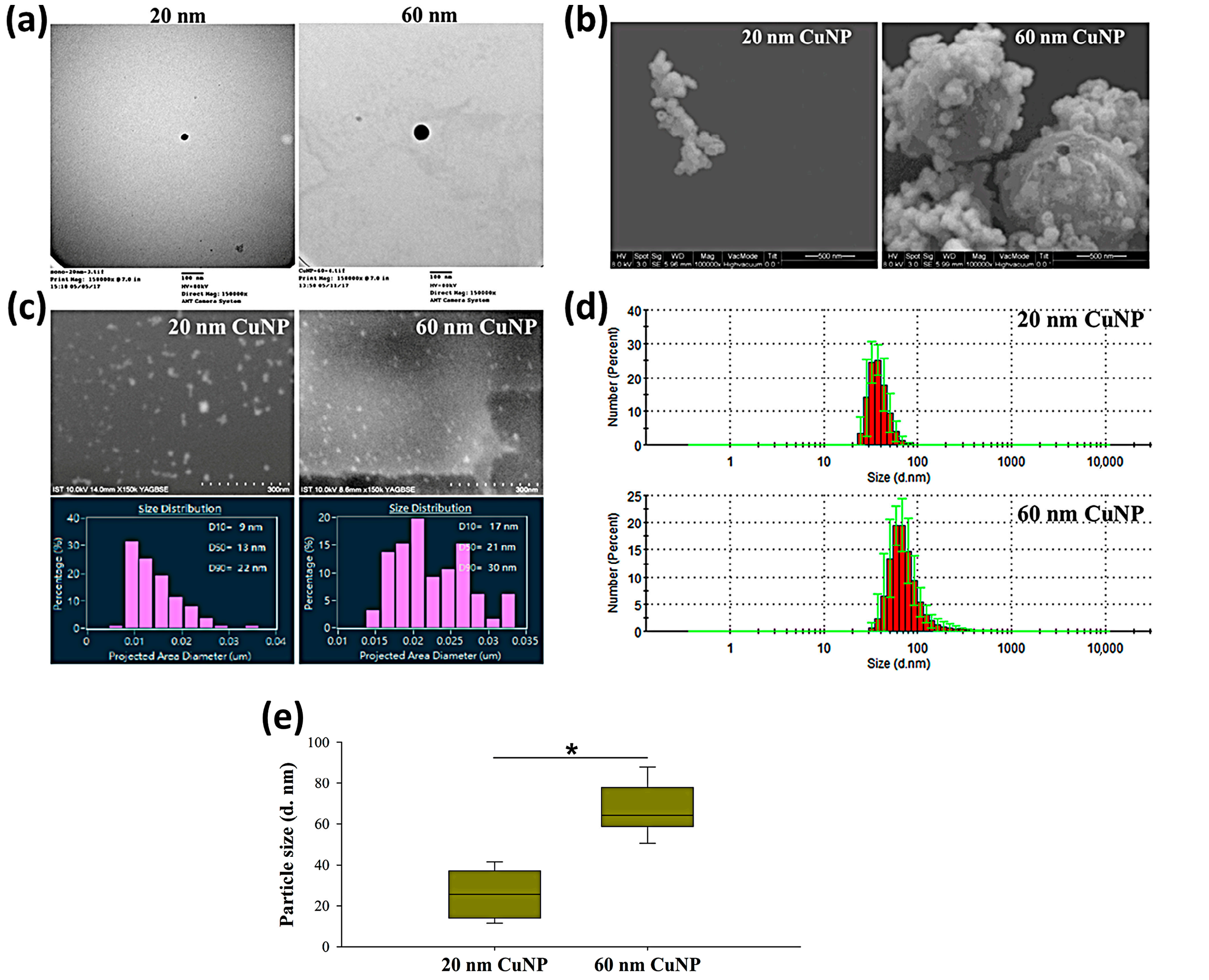

Figure S1. Size determination of CuNPs. The sizes and the shapes of 20 and 60 nm CuNPs were analyzed by TEM (**a**), a liquid particle attractor (**b**), and a Flow AOI (**c**). TEM, liquid particle attractor, and Flow AOI images were obtained with a magnification of 150,000×, 100,000× and 150,000×, respectively. The scale bars indicated 100 nm (a), 500 nm (b), and 300 nm (c), respectively. (**d**) Particle diameters of 20 and 60 nm CuNPs measured by a Zetasizer. (**e**) Statistical analysis of CuNPs particle sizes. Data are presented as mean ± SD from eight independent experiments with triplicates in each

group. The results of particle sizes from (**d**) were analyzed by the one-way ANOVA followed by the Turkey's comparison. Significant differences between groups of 20 and 60 nm CuNPs were set at $p < 0.05$ (*).

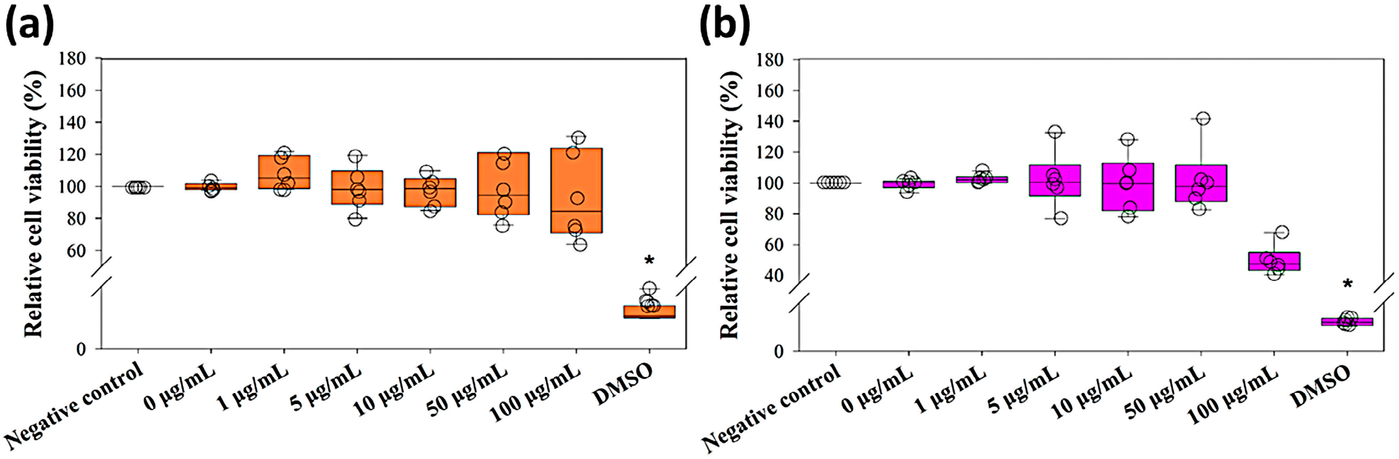

Figure S2. Cytotoxicity of 20 and 60 nm CuNPs in mammalian cells. WS 1 cells were treated with various concentrations (0, 1, 5, 10 , 50, 100 µg/ml) of 20 nm (**a**) or 60 nm (**b**) CuNPs for 24 h. Serum-free medium and pure DMSO were applied in cells as the negative control and the positive control, respectively. Cell viabilities were detected using the MTT assay at the absorbance of 570 nm wavelength. Data were presented as mean ± SD from six independent experiments with triplicates in each group and analyzed by the Kruskal-Wallis-H test, followed with the Dunnett's Method in multiple comparisons versus the negative control. Significant differences were set at $p < 0.05$ (*).

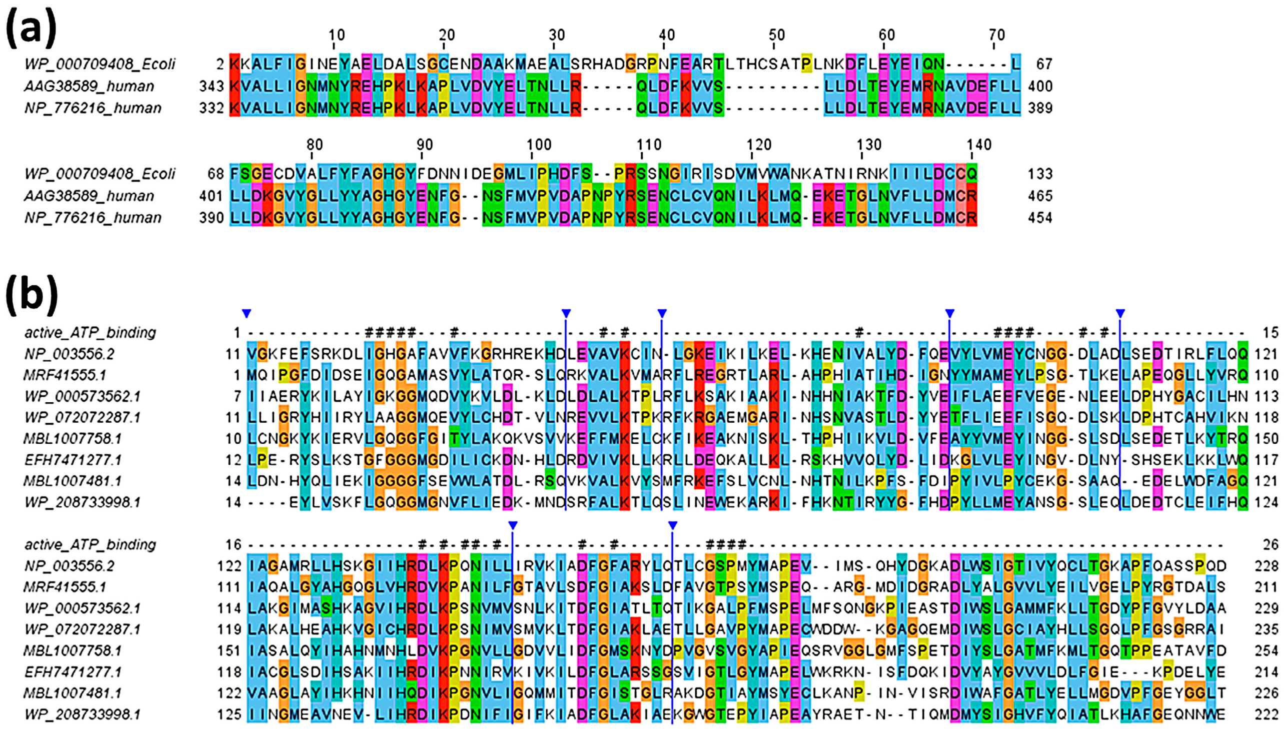

Figure S3. Sequence alignment of modulator active domains in *E. coli*. (**a**) The alignment of the caspase domain (Peptidase_C14 domain, pfam00656) between a caspase protein from *E. coli* (WP_000709408) and two human proteins (NP_776216 and AAG38589). (**b**) The catalytic domain of human threonine-protein kinase ULK1 (NP_003556) aligned with the proteins from *E. coli* identified by NCBI BLASTP. The hash signs (#) marked the possible active and/or ATP binding sites annotated in in STKc_ULK1 domain (cd14202) in CDD database**.** The *triangles* indicates *hidden* regions with divergent alignment. The alignment was generated by Jalview 2**.**
